# Heterotrimeric G Proteins in Plants: Canonical and Atypical Gα Subunits

**DOI:** 10.3390/ijms222111841

**Published:** 2021-10-31

**Authors:** Natsumi Maruta, Yuri Trusov, Alan M. Jones, Jose R. Botella

**Affiliations:** 1 School of Agriculture and Food Sciences, University of Queensland, Brisbane 4072, Australia; n.maruta@uq.edu.au (N.M.); y.trusov@uq.edu.au (Y.T.); 2 Departments of Biology, University of North Carolina at Chapel Hill, Chapel Hill, NC 27599, USA; alanjones@bio.unc.edu; 3 Departments of Pharmacology, University of North Carolina at Chapel Hill, Chapel Hill, NC 27599, USA

**Keywords:** heterotrimeric G proteins, GTPase, signal transduction, GDP-GTP exchange, plant biology, G protein activation, phosphorylation

## Abstract

Heterotrimeric GTP-binding proteins (G proteins), consisting of Gα, Gβ and Gγ subunits, transduce signals from a diverse range of extracellular stimuli, resulting in the regulation of numerous cellular and physiological functions in Eukaryotes. According to the classic G protein paradigm established in animal models, the bound guanine nucleotide on a Gα subunit, either guanosine diphosphate (GDP) or guanosine triphosphate (GTP) determines the inactive or active mode, respectively. In plants, there are two types of Gα subunits: canonical Gα subunits structurally similar to their animal counterparts and unconventional extra-large Gα subunits (XLGs) containing a C-terminal domain homologous to the canonical Gα along with an extended N-terminal domain. Both Gα and XLG subunits interact with Gβγ dimers and regulator of G protein signalling (RGS) protein. Plant G proteins are implicated directly or indirectly in developmental processes, stress responses, and innate immunity. It is established that despite the substantial overall similarity between plant and animal Gα subunits, they convey signalling differently including the mechanism by which they are activated. This review emphasizes the unique characteristics of plant Gα subunits and speculates on their unique signalling mechanisms.

## 1. Introduction

### 1.1. The Classic G Protein Paradigm

**Figure 1:**
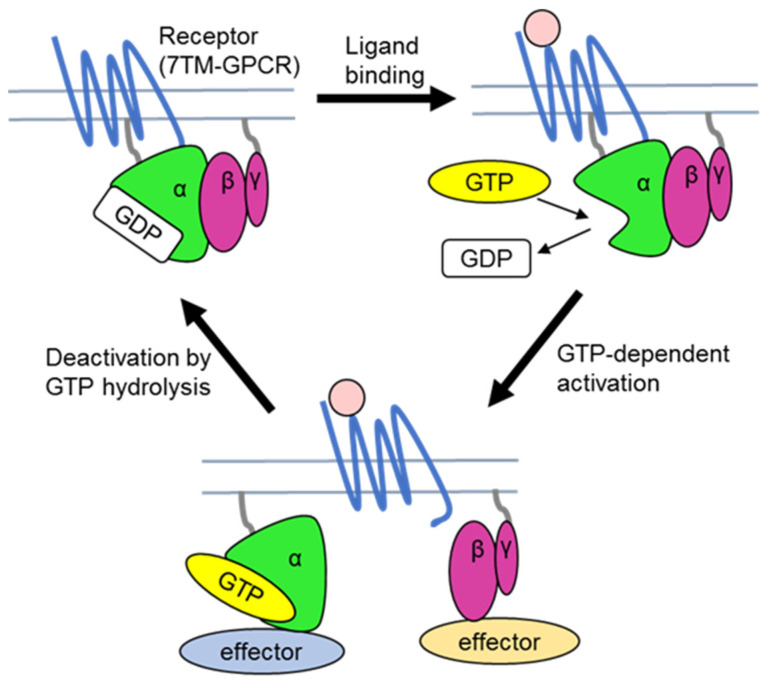
The classic paradigm of heterotrimeric G protein signalling cycle. The heterotrimer consisting of GDP-bound Gα and the Gβγ dimer is associated with a 7TM-GPCR receptor at the plasma membrane in its resting state. Upon ligand binding, GPCR induces a conformational change in Gα, resulting in GDP release followed by GTP binding. GTP-bound Gα separates from Gβγ, and each interact with their cognate effectors to modulate downstream signalling. The intrinsic GTPase activity of Gα leads to GTP hydrolysis, thereby terminating signalling and returning the heterotrimer to the inactive state.

Heterotrimeric GTP-binding protein complexes, minimally comprising Gα, Gβ, and Gγ subunits, mediate the majority of signalling pathways in animals and regulate substantial signalling networks in plants. Their components are found in all major domains of eukaryotic life, placing the origin of the trimeric core structure in the common ancestor of Eukaryotes [1]. The heterotrimeric G protein signalling paradigm, established in animal and yeast cells, states that heterotrimeric G proteins transduce signals from membrane seven-transmembrane-spanning (7TM) G protein-coupled receptors (GPCRs) to downstream cytoplasmic effectors by cycling between active and inactive conformations representing a molecular switch mechanism (Figure 1). The GPCRs and several non-receptor proteins catalyse exchange of guanosine diphosphate (GDP) for guanosine triphosphate (GTP) on Gα, hence known as guanine nucleotide exchange factors (GEFs) [2]. The switch performance depends on binding (on-state) and hydrolysis (off-state) of GTP by Gα subunits. The inactive, GDP-bound Gα is associated with the obligate Gβγ dimer as a complex loosely coupled to a GPCR at the intracellular side of the plasma membrane. Once the GPCR recognises a ligand such as a hormone, neurotransmitter, or peptide, light or volatile, this ternary complex binds more tightly and promotes GDP release, leading to exchange for GTP [3]. GTP-bound Gα undergoes a conformational change that allows heterotrimer dissociation into Gα and the Gβγ dimer. In this active conformation, Gα interacts with specific effectors such as enzymes and ion channels to alter (activate/inhibit) their activities [4]. Gβγ dimers interact with their own effectors to activate specific signalling pathways [5], as well as recruiting modulators to the plasma membrane, specifically to the GPCR. To return the cycle to the resting state, the Gα subunit hydrolyses GTP to GDP at a fixed intrinsic rate, leading to re-association with Gβγ and termination of signal transduction [2,6,7]. The activity of the three core subunits, such as amplitude and duration of the signalling, is regulated by essential regulatory proteins. For example, GTPase accelerating proteins (GAPs), such as regulator of G protein signalling (RGS) proteins, physically bind to Gα and accelerate GTP hydrolysis [8].

### 1.2. The Canonical Gα Subunit Is an Ancient and Conserved Protein

Plant Gα subunits separated from a common eukaryotic ancestor over a billion years ago and have since had an independent evolutionary history. However, to understand it better, we will take a detour in Gα evolution in other eukaryotes. Gα subunits are present in all super groups of eukaryotes, advocating for an early origin and subsequent diversification of these proteins in eukaryotic lineages. Comparative phylogenetic analysis revealed that an entire G protein complex has been lost in many primitive and mostly unicellular eukaryotes, while multicellular eukaryotes possess multiple Gα subunits with greater expansion in opisthokonts (combined animals and yeast groups) [1]. Five major classes of Gα subunits (G_s_, G_i/o_, G_q/11_, G_12/13_, and G_v_) have evolved at the origin of Holozoa (animals and close single-cell relatives), with some classes expanded and others being lost across metazoan phyla [9,10].

Regardless of evolutionary ranks, species can possess only a few or multiple genes for Gα subunits. For instance, the human genome contains 16 genes producing 23 Gα variants through alternative splicing; the amoeba *Naegleria* *gruberi* has perhaps the most—44 distinct Gα subunits [1]—while *Arabidopsis thaliana* (hereafter Arabidopsis) has only four such proteins, three of which are unique to plants. Functional diversification driven by sequence divergence within the superfamily resulted in saltational evolution of these atypical plant Gα subunits [11]. Deviation of the conserved catalytic motifs of some Gα subunits is found in *Dictyostelium* (slime moulds), *Naegleria*, and plants, suggesting reduced levels or complete loss of nucleotide-dependent activity [1,12,13,14]. Some of these Gα subunits also lack the N-terminal residues required for tethering to the plasma membrane, which potentially influences their sub-cellular localization and function.

Plants have two types of Gα subunits: canonical Gα subunits, hereafter GPAs, and atypical, extra-large Gα subunits, hereafter XLGs. While GPAs are conserved, and their ancestry is clearly related to the common Gα ancestor [15], XLGs are structurally distinct and unique to plants [11,14,16]. XLG homologs are present in all major land plant phyla, including the most primitive group of existing land plants, mosses (Bryophyta), placing the origin of XLGs at the dawn of land plants diversification [11,17,18,19]. The ancestral XLG genes diverged substantially from the conserved Gα sequences for a relatively short time, exhibiting traits of saltatory evolution [11]. Most likely, a fitness pressure for rapid adaptation to the new harsh terrestrial environment drove the early evolution of XLG subunits.

## 2. Heterotrimeric G Protein Signalling Components in Plants

Conserved primary sequence motifs and strong interactions among the heterotrimeric G protein subunits allowed identification of plant homologs [20,21,22,23]. Subsequent diversity studies found G protein subunits in all analysed land plants and their closest relatives, charophyte green algae, but not in unicellular algae [17,24,25,26]. Initially, it was assumed that the repertoire of G proteins in plants is limited to the subunits structurally similar to their animal counterparts, or canonical subunits. For instance, Arabidopsis was initially considered to have a single Gα (AtGPA1), a single Gβ (AGB1), and two Gγ subunits (AGG1 and AGG2) [20,21,22,23]. However, the list of G protein subunits was later expanded to include non-canonical proteins unique to plants. Homology searches for Gα-related proteins revealed three extra-large GTP binding proteins, XLGs [16,18]. The C-terminal half of XLGs comprising a Gα-like domain shares evolutionary homology with canonical Gα subunits. The extensive N-terminal domain of XLG proteins has a predicted cysteine-rich motif reminiscent of those found in zinc-finger proteins, a nuclear localization signal (NLS), and a nuclear export signal (NES) [16,18,27]. XLGs localize to the plasma membrane and the nucleus [17,27,28]. Therefore, XLGs were initially considered a novel class of GTP-binding proteins but not a part of the heterotrimeric G protein complex [16,18,29]. Later studies established that XLGs originated from plant Gα subunits and interact directly with Gβγ dimers and RGS, although do so in a nucleotide-independent manner, and participate in Gβγ-dependent signalling pathways [11,12,17,27]. These facts firmly positioned XLGs as Gα subunits of heterotrimeric G proteins, despite their atypical structures [12,17,27]. Comparative genetic studies in Arabidopsis, using Gβ and Gγ knockout mutants, revealed functional inconsistencies, suggesting the existence of an additional Gγ subunit/s [30]. Discovery of the complete set of Gγ subunits in Arabidopsis [31,32,33] set the stage to determine the likely Gγ subunit-based complex compositions. Analyses of Gγ diversity in plants revealed three structural types: (1) type A Gγ subunits share a canonical structure with their animal counterparts; (2) type B Gγ subunits are similar to type A but lack the isoprenylation motif needed to tether the complex to the membrane; and (3) type C Gγ subunits with a cysteine-rich C-terminal tail [25]. As mentioned before, the complex contains only one Gγ subunit, yet all three types interact with the Gβ subunit in the heterocomplex to provide functional selectivity [32,34,35,36,37]. Semi-quantitative yeast-two-hybrid assays revealed preferential binding between Arabidopsis G protein subunits. AtGPA1 shows stronger interaction with AGB1 in the presence of AGG3, while XLG1 and XLG2 prefer AGB1/AGG1 and AGB1/AGG2 dimers [13,17,27]. XLG3 interacts strongly with all three Gβγ dimers [27]. Importantly, the interaction of AtGPA1 and XLG3 with AGB1/AGG3 is competitive [27], suggesting that XLGs bind the same Gβγ interface as the canonical Gα. A fourth component of the Arabidopsis complex is AtRGS1, regulator of G protein signalling 1. AtRGS1 accelerates the intrinsic GTPase activity of Gα subunits in land plants and exhibits structural dissimilarities to their animal counterparts [38,39,40]. Unlike RGS proteins in animals, the single-copy Arabidopsis AtRGS1 contains seven putative transmembrane domains [38]. Interestingly, AtRGS1 internalizes through endocytosis upon treatment with glucose [41], displaying a receptor-like behaviour, although direct perception of glucose (or a sugar metabolite) by AtRGS1 has not been shown. Moreover, genetic ablation of *AtRGS1* does not confer the most marked G-protein subunit phenotypes (e.g., *agb1* null phenotypes, [42,43,44]) under static conditions, but do so under dynamic conditions [45,46]. Loss of AtRGS1 does not eliminate G protein-mediated hormone responses [26]. Thus, AtRGS1 is currently considered a dynamic signal modulator rather than a receptor, per se [47,48].

While the components of the heterotrimeric G protein complex core have been established [49], the search for genuine GPCRs (i.e., 7TM proteins providing GEF activity) has yielded none so far [50]. Several candidate proteins with predicted seven transmembrane domains were considered for the role [51,52], but thorough analyses discarded them [50,53,54].

## 3. Gα Subunit Prerequisites for Nucleotide Exchange

**Figure 2:**
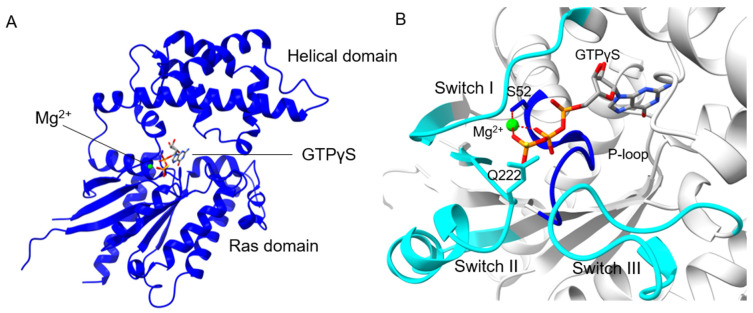
The structure of the Arabidopsis canonical Gα subunit (AtGPA1) in its GTPγS-bound form. (**A**) Overall tertiary structure of AtGPA1 (PDB: 2XTZ), showing the Ras domain and the helical domain. (**B**) Close-up view on the nucleotide-binding pocket with three switch regions highlighted in cyan and the G1 box (also known as the P-loop) highlighted in blue. Side chains of Ser 52 (P-loop) and Gln 222 (Switch II) are shown. The Mg^2+^ ion coordinates the side chain of Ser 52 and the β- and γ-phosphate moieties of GTP, indicated in red lines (dash). The side chain of Gln 222 hydrogen bonds with two water molecules providing hydrolysis of GTP.

The common feature of the superfamily of GTPases is their ability to bind and hydrolyse GTP, providing a molecular switch function [55,56]. The primary sequence of a typical GTPase contains five highly conserved motifs, termed G1 to G5 boxes, which form distinctive loops on the protein tertiary structure and are necessary for GTP binding and hydrolysis [57]. These boxes are present in all members of the GTPase family, including AtGPA1, and form a structurally conserved Ras-like domain (Figure 2A). The G1 box, also known as the P-loop, has a consensus sequence of GXXXXGK(S/T) (single-letter amino acid code with X standing for any amino acid) and coordinates the α- and β-phosphates of GTP (Figure 2B). Notably, in conventional Gα subunits, the G1 consensus is more stringent G(A/T/Q/P)G(E/D)SGK(S/T) [1,13]. The G2 box is responsible for cofactor Mg^2+^ coordination. The DXXGQ motif of the G3 box binds the γ-phosphate of GTP and Mg^2+^. The (N/T)KXD motif of the G4 box is important for guanine ring stabilization in the pocket. Finally, the G5 motif (T/G)(C/S)A interacts with the guanine base. GTP binding causes a conformational switch in three specific segments of Gα. These segments are therefore termed Switches I, II, and III (Figure 2B). These conformational changes reduce the area of the buried interface of Gα with Gβγ [3,57]. Compared to small GTPases, Gα subunits also contain a unique helical domain (Figure 2A) that consists of approximately 120 amino acids and is located between the G1 and G2 boxes [58]. Little was known of the function of this domain, until comparative structural studies of the helical domains of human Gα_i1_ and Arabidopsis AtGPA1 in domain swap experiments showed that this domain controls the intrinsic GDP-GTP exchange rate and protein stability [59,60]. More specifically, in both Gα_i1_ and AtGPA1, the guanine nucleotide is bound within the Ras domain and is buried under the helical domain, where mobility of the helical domain provides an opportunity for the nucleotide release. In animals, GPCRs catalyse nucleotide release by rearrangement of the Ras domain [61]. This process is the slowest in the nucleotide-exchange cycle and therefore determines the rate of the signalling turnover in animal G proteins [61,62]. In plants, the helical domain is more mobile and GDP release occurs spontaneously, without help of a receptor [59,60], changing the cycle limiting step to GTP hydrolysis [39].

## 4. GTP Requirements in Plant G Signalling

Arabidopsis AtGPA1 displays high structural similarity to animal Gα_i_ in the tertiary structure with an overall RMSD of 1.8 Ȧ [59] and accepts nucleotide-dependent conformations balanced by interactions with AtRGS1 and/or phospholipase Dα1 [38,39,63]. However, biochemically, AtGPA1 is different from its animal counterparts, binding GTP very fast (k_obs_ = 1.4–4.4 per min, depending on evaluation method) compared to human Gα_oA_ (k_obs_ = 0.09 per min) [39]. GTP hydrolysis by AtGPA1 is one of the slowest for GTPases (K_cat_ = 0.12 per min) [39]. These unusual biochemical properties of plant canonical Gα subunits result in a quite non-canonical nucleotide-exchange cycle with GTP hydrolysis, rather than GDP release, being the rate-limiting step [39,64]. Despite clear biochemical evidence for G cycling in vitro, the GDP- and GTP-bound states of AtGPA1 might be indistinguishable, as it targets the plasma membrane before the trimer formation, and its constitutively active form, AtGPA1(Q222L), is bound to the Gβγ dimer [13,65], challenging the role of G cycling in plant signalling. Adding to the challenge, the aforementioned XLGs have low or no affinity to GTP and can work without it. Analyses of a wide range of XLGs from various plants revealed very weak conservation within the nucleotide-binding motifs and apparent losses of crucial amino acids and entire motifs [14,16]. Confirming this notion, non-quantitative [11,14,16,66] and quantitative in vitro assays showed that XLGs have very low steady-state GTPγS binding, up to two orders of magnitude lower than that of AtGPA1 [12]. Given such low affinity for guanine nucleotide, and taking into consideration the estimated concentration of guanine nucleotide in plant cells, XLG2 is likely to exist as a nucleotide-free protein in vivo [12], and as such, it must function in a GTP-independent manner. This hypothesis was supported by genetic experiments showing that Arabidopsis XLG2 mutated to lose GTP-binding activity is still able to function normally in various aspects of plant defence and development [14].

As discussed above, the plant Gα has canonical structure and is capable of performing the nucleotide-exchange cycle; however, there are observations that suggest that, in plants, canonical Gα might be able to function in a nucleotide-independent manner. Mutation at G3, substituting terminal glutamine with lysine (Q->L substitution), results in the inability of canonical Gα subunits (in plants and animals) to hydrolyse bound GTP and thus provides constitutively active Gα subunits [67,68]. In humans, analogous mutations are associated with GH-secreting pituitary tumours and thyroid tumours [69,70], resulting in the elevation of cellular cAMP (in this case, over-activation), contributing to abnormal cell growth [71]. In plants, similar mutations confer variable behaviour. For example, rice *dwarf 1*, *d*1, a null mutation in the Gα gene, *RGA1*, produces shorter grains compared to those of wild-type rice plants [44,68,72,73]. Expression of RGA1(Q223L) results in longer grains than grains of wild type [68], which is consistent with constitutive activation of the signalling pathway mediating this trait. In Arabidopsis, ectopic expression of AtGPA1(Q222L) produces longer etiolated hypocotyls, longer primary roots [38,74], and increased stomatal density relative to wild type [75], which is also consistent with constitutively activated signalling. On the contrary, expression of RGA1(Q223L) complements the *d*1 dwarf phenotype only to wild-type height instead of producing taller plants [68], which is inconsistent with the constitutive activation of RGA1-mediating signalling and raises the question of whether this function requires GTP-binding and nucleotide-exchange. In maize, knockout of the Gα subunit compact plant 2 (CT2) also causes dwarfism, shortened leaves, and enlarged shoot apical meristem [76]. Attempts to complement these phenotypes with CT2(Q223L) achieved only partial complementation, while wild-type CT2 protein fully rescues all the mutant phenotypes [77], leading to the conclusion that CT2(Q223L) functions as a weak, rather than a constitutively active, allele [77]. Direct genetic complementation studies on *gpa1* mutants expressing nucleotide-free AtGPA1(S52C) showed complementation of multiple *gpa1* mutant phenotypes, including reduced rosette and flower size, rounder leaf shape, flattened silique tips, shorter petioles, and etiolated hypocotyls [13]. Expression of WT and constitutively GTP-loaded AtGPA1(Q222L) also complemented the phenotypes. Most importantly, nucleotide-free AtGPA1(S52C) was not able to complement all *gpa1* mutant phenotypes, indicating the existence of nucleotide-dependent and nucleotide-independent G-protein signalling pathways. Moreover, GTP-bound AtGPA1(Q222L) and nucleotide-free AtGPA1(S52C) interacted with Gβγ1 and Gβγ2 dimers *in planta* with similar strength, suggesting nucleotide exchange-independent heterotrimer formation. Even though the possibility that the AtGPA1(S52C) mutation could adopt a stable on-state conformation cannot be discarded [78], the fact that AtGPA1(S52C) failed to complement all *gpa1* mutant phenotypes [13] argues that signal discrimination and activation does not always require G cycling, as will be discussed below in Section 8, entitled *Plant Gα Proteins in Signalling Models*.

Classically, GTP binding causes Gα to change conformation leading to heterotrimer dissociation [7]. Thus, dissociation may be used as an indirect indicator of heterotrimer activation. Some heterotrimers, however, do not dissociate upon GTP binding but undergo structural rearrangements [79,80,81]. In rice, Gα subunit (RGA1) bound to non-hydrolysable GTPγS as well as mutated RGA1(Q223L) exist as a free form [34]. In maize, mutated Gα CT2(Q223L) does not interact with the Gβγ dimer [77] supporting the dissociation model. On the contrary, the Arabidopsis AtGPA1(Q222L) interacts with Gβγ, suggesting variability of the activation models for plant G proteins [13,65]. Curiously, Arabidopsis G proteins are part of larger protein complexes (~700 kDa), and treatment with GTPγS promotes only partial dissociation of AtGPA1 from the complex [82]. These observations do not support the GTP binding dependency for Gα-mediated signalling, but rather advocate the hypothesis that AtGPA1 functions through both nucleotide exchange-dependent and -independent mechanisms. The existence of nucleotide exchange-independent function/s of plant ancestral Gα could explain the origin and evolution of XLGs, which became specialised for nucleotide-independent roles and eventually lost their ability to cycle guanine-nucleotides.

## 5. Physiological Roles of XLGs

Characterization of Arabidopsis XLG-deficient mutants revealed diverse functional roles of XLGs, including root development [18,83], stomata development [27], flowering [66], stamen development [84], sensitivity to hormones and sugar [18,27,83,84], and defence against pathogens [17,85,86]. A study on an Arabidopsis quadruple mutant lacking the canonical AtGPA1 and all three XLGs uncovered that these four Gα subunits function cooperatively or antagonistically in developmental processes [11]. A particularly interesting observation is the involvement of XLG2 in plant basal immunity in Arabidopsis [17,86] with specific signalling components recently unravelled [14,85,87]. Functional analysis of a sole XLG subunit from moss *Physcomitrella patens* revealed its involvement in gametophyte development, sporophyte formation, and thus completion of its life cycle [19]. Interestingly, the *P. patens* genome lacks a canonical Gα-encoding gene, while it possesses genes for Gβ and Gγ subunits, suggesting that the XLG subunit fulfils the role of Gα [19]. Furthermore, a recent study characterized XLGs in maize, revealing that they regulate early plant development functioning redundantly with the canonical Gα [77]. Notably, knocking out three maize XLGs resulted in hyper-activation of cell death response and seedling lethality [77].

The initial observation that Arabidopsis *XLG2* and *XLG3* transcriptional activation was induced by *Pseudomonas syringae* pv. *tomato* (*Pst*) DC3000 prompted researchers to study their role in plant defence [86]. Reverse genetic analysis revealed that the *xlg2* mutant was more susceptible to *Pst* DC3000, *Pst* DC3000 *avrRPM1*, and *P. syringae* pv. *phaseolicola* than wild-type plants. At the same time, *xlg1* and *xlg3* mutants were indistinguishable from wild-type plants in susceptibility. Intriguingly, interaction between XLG2 and AGB1 was detected by co-immunoprecipitation assays in infected leaves, but not in control, while yeast-two-hybrid (Y2H) assays failed to detect the interaction [86]. While AGB1 provides resistance against the necrotrophic fungal pathogens, *Botrytis cinerea* and *Alternaria brassicicola*, all three *xlg* single mutants behaved similarly to the wild type in response to these pathogens [86]. A follow-up study reported that an *xlg* double knockout mutant, *xlg2 xlg3*, displayed similar levels of susceptibility to *agb1* or *agg1 agg2* mutants upon infection with *Fusarium oxysporum* and *A. brassicicola* [17]. Importantly, a quadruple mutant lacking AGG1, AGG2, XLG2, and XLG3 was susceptible to these pathogens at levels similar to *agb1*, *agg1 agg2*, and *xlg2 xlg3* mutants, revealing no additive effect and thereby indicating that XLG2/3 and Gβγ1/2 mediate the same immune signalling pathway [17]. It is noteworthy that, while XLG2 and AGG1 are major contributors to defence responses, XLG3 and AGG2 play supporting roles, conditionally complementing the lack of XLG2 and AGG1, respectively. Interaction between XLGs and AGB1 requires presence of an AGG subunit [17,27]. The XLG-Gβγ interaction occurs at the plasma membrane, not in the nucleus, although all three subunits are able to localize to the nucleus individually when ectopically expressed.

## 6. Receptor-Like Kinases (RLKs) May Provide the Signal Discrimination Compensating for the Lack of GPCRs

Given that: (1) GTP binding is not the rate-limiting step in plant G cycling, (2) loss of a GPCR-like RGS protein does not ablate signal transduction, (3) phosphorylation modulates the activity of plant G signalling [41,88], and (4) canonical Gα and XLGs can act independently of nucleotide-exchange, it is conceivable to hypothesize that G cycling is activated through phosphorylation by non-GPCR-like receptors. A large family of single-transmembrane receptor-like kinases (RLKs) that play crucial roles in a variety of G protein-mediated responses including innate immunity, where RLKs serve as receptors for various pathogen-associated molecular patterns (PAMPs). PAMPs such as flg22 and elf18 induce expression of genes encoding G protein subunits (*AGB1*, *XLG2*, and *XLG3*) [17,86]. PAMP-induced disease resistance to *P. syringae* and *Agrobacterium tumefaciens* was strongly attenuated in *agb1* and *xlg* mutants compared to the wild type [14,17,85,89,90]. Reactive oxygen species (ROS) production induced by PAMPs is one of the earliest defence responses. ROS serve as antimicrobial compounds, cross-link plant cell walls, regulate callose deposition, and act as a signal to induce stomatal closure and immune responses in neighbouring leaves [91]. ROS generation requires the activity of plasma membrane-localized NADPH oxidases, known as respiratory burst oxidase homolog (Rboh) proteins. Particularly, RbohD is responsible for ROS production during pathogen attack. RbohD-mediated ROS production is dependent on phosphorylation by a receptor-like cytoplasmic kinase (RLCK), botrytis-induced kinase 1 (BIK1), calcium-dependent protein kinases (CDPKs), and Ca^2+^ binding [91]. Mutants deficient in XLG2/3, AGB1, and AGG1/2 are significantly impaired in ROS production upon treatment with flg22, elf18, and chitin [14,17,54,85,87,89,90,92]. XLG2/3 physically interact with RbohD regardless of flg22 treatment [85]. The *gpa1* mutant showed only a slight reduction [93] or no difference [89,92] in ROS production upon PAMP perception compared to wild-type plants. Yet, AtGPA1 and RbohD constitutively interact with each other [94]. Analysis of a triple mutant lacking AGB1, RbohD, and RbohF demonstrated that the *agb1* mutation suppresses the enhanced disease resistance displayed by *rbohD rbohF* double mutants in response to *Pst* DC3000 and the oomycete *Hyaloperonospora arabidopsidis*, while response to *Plectosphaerella* *cucumerina* was not affected [92]. Thus, depending on the pathogen, AGB1 and RbohD/F can act either cooperatively or independently. This study also showed that salicylic acid (SA)-mediated responses do not involve AGB1 but include both RbohD and RbohF during *Pst* DC3000 infection [92]. All the above-mentioned findings indicate that G proteins are involved in RLK-mediated immunity.

PAMPs such as flg22, elf18, and chitin activate defence-related MAP kinases, MPK3, MPK4, and MPK6, causing their phosphorylation. It has been reported that flg22, elf18, and chitin activate all three MPKs in *agb1* and *xlg2 xlg3* mutants [93], although MPK4 activation is somewhat weaker in *agb1* plants [89], suggesting that G proteins are not involved in this process. However, a different group observed that phosphorylation of all three MPKs is defective in *agb1* mutants [95,96]. These apparently contradicting results could be explained by differences in flg22 concentration used in the studies, with high elicitor concentration (1 µM) inducing all MPKs in wild-type plants and *agb1* mutants to similar levels [93], while lower concentration (100 nM) allowed us to distinguish between wild-type plants and G protein mutant responses [95,96]. It is well established that high and low ligand concentrations can activate different signalling pathways [97]. PAMP-triggered expression of defence marker genes was compromised in *agb1* and *xlg2 xlg3* mutants, providing additional evidence for association between RLKs and G proteins [87,95].

Constitutive activation of immune response reduces plant fitness by inhibiting plant development; therefore, immune signalling pathways are under tight regulation and activated only upon pathogen recognition [98]. One of the negative regulators of immune signalling pathways is an RLK named BAK1-interacting receptor-like kinase1 (BIR1). Knockout of *BIR1* results in constitutive activation of immune responses, leading to seedling lethality, as the *bir1* mutant has increased SA levels, *PR1/PR2* expression, and H_2_O_2_-induced cell death [99]. A search for suppressors of the *bir1* lethal phenotype led to the identification of *phytoalexin deficient 4* (*PAD4*), *suppressor of BIR1* (*SOBIR1*), and G protein-encoding genes (*AGB1/AGG1/AGG2/XLG2*), as mutations in these genes partially rescued the *bir1* phenotype [17,89,99]. Interestingly, *gpa1* mutations did not alter the *bir1* phenotype [89], further confirming that the canonical Gα is not a partner of Gβγ in this process.

## 7. RLKs/RLCKs Physically Interact with and Phosphorylate G Protein Subunits

In addition to the functional links between RLKs/RLCKs and G proteins described above, there is an increasing amount of evidence demonstrating direct physical interaction between heterotrimeric G protein subunits and defence-related RLKs. AtGPA1, AGG1, and AGG2 interact with BAK1, BIR1, and CERK1 in split-ubiquitin and BiFC assays [100], while XLG2 displayed interaction with the FLS2/BIK1 complex as well as with BIR1 [14,85]. Interestingly, the interaction between XLG2, FLS2, and BIK1 is dynamic, with flg22 treatment causing dissociation of both XLG2 and BIK1 from the receptor [85]. Additionally, FLS2 and BIR1 interact with XLG2 competitively, suggesting that these kinases bind the same XLG2 surfaces, while FLS2 and BIK1 do not compete for XLG2 [14], supporting the idea that XLG2 interacts with the FLS2/BIK1 complex.

LC–MS/MS analysis of XLG2 derived peptides obtained after flg22 treatment revealed that XLG2 is phosphorylated in its N-terminal domain. Further analyses identified that BIK1 can phosphorylate XLG2 in vitro [85]. Importantly, the established phospho-sites were found to be necessary for the functionality of XLG2 [85]. Protein metabolism assays show that interaction with XLG2 and AGB1 protects BIK1 protein from degradation by E3 ligases, PUB25, and PUB26 [85,101].

AtRGS1, a modulator of the AtGPA1 signalling cycle, also physically interacts with BAK1, BIR1, FLS2, and LYK5, which is a co-receptor of CERK1 [87,102]. BAK1 phosphorylates the Ser 428 residue of AtRGS1, and flg22 treatment promotes such phosphorylation and subsequent endocytosis of AtRGS1 in a FLS2-dependent manner [47], suggesting that the flg22-induced AtRGS1 internalization leads AtGPA1 to spontaneously self-load GTP. Multiple RLKs include BAK1 phosphorylate AtGPA1 [48]. The specific phosphorylation of AtGPA1 on the Tyr 166 residue, which requires BAK1, although not directly, increases binding with AtRGS1 in a GDP-bound state, unlike the typical mechanism, where they interact in the transition state with GDP + AlF [48]. Overall, Tyr 166 phosphorylation substantially slows down the AtRGS1-accelerating GTPase activity of AtGPA1 [48]. Curiously, it was proposed that flg22-induced activation of G proteins within the FLS2/AtRGS1 complexes, involving both XLG2 and AtGPA1 modules, occurs in a GTP-dependent manner [87]. Although this might be true for AtGPA1, extending the conclusion for XLG2 is tenuous. For instance, this report assumes that XLG2 self-loads GTP similarly to AtGPA1, and that AtRGS1 enhances XLG2 GTPase activity [87]. Biochemical evidence shows that XLG2 has much lower affinity for GTP and is probably nucleotide-free in vivo [12]. Secondly, although AtRGS1 binds XLG2, it was not demonstrated that it regulates GTP hydrolysis by XLG2 [12]. Additionally, coupling between AtRGS1 and XLG2 might be conditional, since it was not detected in other studies [11].

## 8. Plant Gα Proteins in Signalling Models

**Figure 3:**
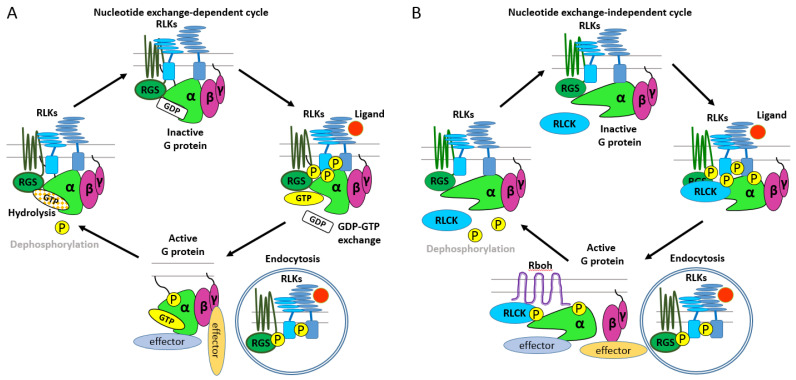
Model for heterotrimeric G protein signalling in plants. (**A**) Nucleotide exchange-dependent cycle. The heterotrimer consisting of GDP-bound canonical Gα and the Gβγ dimer is associated with a 7TM-RGS and RLKs at the plasma membrane in its resting state. Upon ligand binding, RLKs phosphorylate Gα and RGS. Ligand binding is followed by receptor and RGS endocytosis and subsequent de-repression of Gα, which releases GDP and binds GTP. GTP-bound Gα does not necessarily separate from Gβγ, and each of the two components modulate downstream signalling cascades. Dephosphorylation (research is urgently needed) and binding to a new RGS leads to GTP hydrolysis terminating signalling and returning the heterotrimer to the inactive state. (**B**) Nucleotide exchange-independent cycle. The heterotrimer consisting of either canonical Gα or XLG and the Gβγ dimer is associated with a 7TM-RGS (the role of RGS here is yet to be determined), RLKs, and RLCKs at the plasma membrane in its resting state. Upon ligand binding, RLKs phosphorylate Gα/XLG, RGS, and RLCKs. Ligand binding is followed by receptor endocytosis. Here, activation could be caused by de-repression or phosphorylation-mediated activation of Gα/XLG or Gβγ (the activation mechanism is not established). In case of Arabidopsis, XLG2 dissociates from Gβγ upon perception of flg22 [85]. Both Gα/XLG and Gβγ modulate downstream signalling cascades. One example of effector activation is the BIK1/XLG2-mediated activation of RbohD [85]. Hypothetically, dephosphorylation (research is urgently needed) leads to signal termination and reassociation of the heterotrimer into the inactive state.

Bearing in mind the idiosyncrasies of plant G proteins, we conclude that their activation mechanism does not fit the classical G protein paradigm, whether it is canonical nucleotide-dependent GPAs or unconventional nucleotide-independent XLGs. In general, molecular switches, such as G proteins, initiate downstream signalling in three distinct modalities: (1) activation, (2) derepression, and (3) concerted (a combination of activation and derepression) [103]. One of the first models for a nucleotide-exchange dependent mechanism of action of plant canonical GPA1 was based on derepression [40]. The mechanism considered absence of GPCR, spontaneous uptake of GTP molecule, and GAP activity of RGS1. It was suggested that RGS1 constitutively enhancing Gα intrinsic GTPase activity holds Gα in its off-state. Glucose supplement causes endocytosis of AtRGS1, leading to its mechanistic uncoupling from AtGPA1 [41]. Thus, the derepressed AtGPA1 rapidly exchanges GDP for more abundant GTP and accepts the active conformation [40,41]. This model was supported for another ligand, flg22, and expanded for XLG2 [87]. However, nucleotide-exchange by XLG2 is a questionable assumption. The model was adjusted by addition of RLKs/RLCKs, with no Lysine 8, AtWNK8, and BIK1, and phosphorylation-mediated regulation of the AtRGS1 [41,87]. This model was recently revised and updated with a switch mechanism consisting of four stages determined by phosphorylation/dephosphorylation of Gα and the nucleotide (GDP or GTP) bound [45]. Several reports hypothesized that phosphorylation plays a significant part in plant G protein activation mechanisms [104,105,106,107,108]. Taking together the experimental data and reported assumptions, we generated a hypothetical model of the nucleotide exchange-dependent activation cycle for plant G proteins (Figure 3A).

While canonical Gα exploit both nucleotide exchange-dependent and -independent activation mechanisms [13], XLGs seem to perform only in a nucleotide-independent manner [14]. Both canonical Gα and XLGs may also buffer/augment each other cycles through competing interactions with the Gβγ dimer. The four-state model by Ghusinga et al. has not yet incorporated the XLG proteins [45]. It is tempting to speculate that XLGs run through on-state/off-state cycles by phosphorylation/dephosphorylation activities of specific kinases and phosphatase, respectively (Figure 3B). However, direct evidence showing that phosphorylation of an XLG turns on or off a signalling cascade is missing. At the same time, while phosphorylation for AtGPA1 and XLGs is established [48,87,88,109], de-phosphorylation of G protein subunits has not been reported to the best of our knowledge.

## Data Availability

Not applicable.
